# Zinc, Magnesium and Vitamin K Supplementation in Vitamin D Deficiency: Pathophysiological Background and Implications for Clinical Practice

**DOI:** 10.3390/nu16060834

**Published:** 2024-03-14

**Authors:** Andrius Bleizgys

**Affiliations:** Clinic of Internal Diseases and Family Medicine, Institute of Clinical Medicine, Faculty of Medicine, Vilnius University, 08661 Vilnius, Lithuania; andrius.bleizgys@gmail.com

**Keywords:** vitamin D, zinc, magnesium, vitamin K, supplementation

## Abstract

Zinc, magnesium, and vitamin K are important nutrients for humans. There are various factors that contribute to the development of their deficiency, which might result in or exacerbate various diseases. These nutrients can also interact with vitamin D metabolism and activity. This review discusses the main aspects of zinc, magnesium and vitamin K metabolism and action in the body, their clinical significance, and the “crosstalk” with vitamin D, as well as providing general suggestions for clinical practice when supplementation with these nutrients might be useful, in addition to vitamin D supplementation.

## 1. Introduction

Vitamin D (VitD) and its role in human health are still among the main topics in the scientific literature as well as in the mass media. It is well known that VitD has a pleotropic activity in human organisms, and many observational studies showed associations between low VitD and various illnesses [1,2]. The high incidence of VitD insufficiency around the world is concerning and calls for efforts to ameliorate the situation [2]. Many guidelines for low VitD status prevention and treatment were developed during the last few decades [2,3,4,5,6,7,8,9,10,11]. Moreover, some of them already considered the potential extra-skeletal benefits of VitD and therefore recommended both to achieve and to maintain higher levels of serum 25-hydroxy vitamin D (25OH-D; a marker of VitD status)—at least 75 nmol/L, identified as “sufficient” levels regarding not only musculoskeletal health. However, many (but not all) interventional studies in humans failed to confirm significant benefits for various health parameters of VitD supplementation, even though many participants in those trials were able to improve their VitD status (i.e., to achieve the “sufficient” serum 25OH-D levels). These failures might be explained by improper trial design, as discussed elsewhere [12,13,14,15,16]: too small sample size; too short duration of the trial; inappropriate VitD dose; wrong selection of participants (e.g., those having initially “sufficient” serum 25OH-D levels are potentially not expected to receive benefits from additional VitD supplementation; similarly, those with irreversible anatomical changes to the organs in the case of advanced disease were included in a trial). The different personal vitamin D response indexes of the trial’s participants might also be an explanation [17].

However, some other reasons might have been underestimated. It is well known that the metabolism and the levels of different VitD metabolites, in particular the calcitriol levels (calcitriol = active or hormonic form of VitD) are influenced by numerous substances—parathormone (PTH), fibroblast growth factor-23, serum calcium (Ca) levels, magnesium (Mg), etc. Interestingly, zinc (Zn) and vitamin K (VitK) can also have some “crosstalk” with VitD; however, this is less discussed in the scientific literature in comparison to, e.g., PTH’s or Mg’s roles. Some of the [2,3,4,5,6,7,8,9,10,11] VitD guidelines also include brief recommendations on Mg intake; none of them, however, discuss the importance of VitK or Zn regarding VitD. Nonetheless, it could be speculated that the undervalued (unsuspected, undiagnosed, and, consequently, untreated) insufficiency of nutrients like Mg, Zn, and, to some degree, VitK could weaken the action of calcitriol in various organs and tissues. This may also explain why many clinical trials did not show the beneficial outcomes that had been initially presumed, and, in some cases, why adverse reactions (e.g., calcification of soft tissues) developed after supplementation with VitD. Of note, the deficiency of various nutrients (including Zn, VitK and Mg), also called “the hidden hunger” is widespread in many countries and might lead to serious illnesses [18].

The current narrative review outlines the main pathophysiological aspects of the impact of Zn, Mg and VitK on the human body in general and on VitD metabolism and activity in particular, as well as providing some insights for clinical practice.

## 2. Magnesium

Normally, an adult’s body has about 24 g of Mg, the majority of which (99%) is found in the bones, muscles, and soft tissues. Extracellular fluids contain only 1% of the body’s total Mg [19]. Mg is the second most prevalent cation inside cells and the fourth most abundant cation overall in the human body [20]. 

The primary nutritional sources of Mg are the following: whole grains, dark green vegetables, legumes, nuts, seeds, and seafood [21,22]. In the small intestine, only around 30% of the total dietary Mg is absorbed; however, in the case of deficiency, increased absorption may occur through passive diffusion or a saturable transport mechanism. The kidney primarily controls the excretion of Mg, with the glomerulus filtering 80% of the total plasma Mg. The renal tubules reabsorb the filtered Mg: 10–15% in the proximal tubule, 60–70% in the thick ascending limb, and 5% in the distal tubule, respectively [19].

Mg functions as a structural or catalytic component of enzymes, as well as of substrates, in hundreds of enzymatic processes. It is involved in numerous processes—ion channel activity and signal transduction, stabilization of membranes, aerobic and anaerobic metabolism, proliferation, differentiation and apoptosis of cells, and angiogenesis [19,21,23]. The potentially beneficial impact of Mg on glucose and lipid metabolism, innate and adaptive immunity, and the nervous system deserves particular attention [20,21,24,25,26]. A deficiency of Mg is associated with headaches, hyperemotionality, generalized anxiety, insomnia, asthenia, depressive states, muscle weakness, numbness and cramps, exacerbations of bronchial asthma, increased risk of stroke, progression of diabetes mellitus and congestive heart failure, worse control of arterial hypertension, higher risk of cardiac arrythmias, alterations in blood lipids and progression of atherosclerosis, severe forms of infectious diseases, long-term COVID-19 syndrome, increased risk of cancers, and many other illnesses [20,21,22,23,24,25,26,27].

For normal VitD metabolism and activity, an optimal Mg status is required. It is known that Mg acts as a cofactor in various processes, such as VitD binding to VitD-binding protein, 25OH-D synthesis, calcitriol synthesis, activity of 24-hydroxylase (i.e., the enzyme, deactivating 25OH-D and calcitriol), and VitD receptor (VDR) expression for cellular effects [19]. Low Mg levels enhance the release of PTH, but very low Mg concentrations suppress the secretion of PTH [28]. Thus, indirectly, severe Mg deficiency could also contribute to reduced renal synthesis of calcitriol, since PTH is known to stimulate renal 1α-hydroxylase. Mg deficiency notably influences the VitD levels in people with a high risk of low VitD, such as women, non-Hispanic African Americans, obese people, or persons with the highest levels of PTH in the blood [28].

On the other hand, VitD could itself impact Mg metabolism. The majority of Mg is absorbed regardless of VDR or VitD; nevertheless, calcitriol can increase Mg absorption in the gut by upregulating intestinal VDR, and this contributes to maintaining Mg homeostasis [19,22,27,28]. Interestingly, VitD can increase renal Mg excretion and therefore decrease Mg retention [29]; possibly, this is realized via PTH suppression (by calcitriol), since PTH contributes to renal Mg reabsorption [27]. 

The serum Mg levels are not a good indicator of the intracellular or whole-body Mg content, since the blood serum contains just 0.3% of the body’s total Mg [19]. Nevertheless, in clinical practice, the serum Mg levels usually serve as an indicator of Mg status [27,30]. Mg^2+^ serum levels of 0.7–1.1 mmol/L (or of 0.75–0.95 mmol/L, as recommended by others) are considered “normal”, and serum Mg^2+^ values less than 0.7 mmol/L are generally considered as hypomagnesemia [19,22,23]. It was suggested that for better Mg status assessment, the renal Mg^2+^ excretion should also be evaluated. Serum Mg^2+^ levels below 0.82 mmol/L and Mg^2+^ urinary excretion of 40–80 mg/day can indicate Mg deficiency [23]. Other methods, e.g., the Mg load test, total RBC Mg assay, ionized RBC Mg assay, isotopic analysis of Mg, and intracellular electrolytes and magnesium test, are rarely used due to their high cost and/or limited diagnostic usefulness for certain patient groups [22,27,28,31].

Many risk factors can contribute to the development of Mg deficiency, including:

- insufficient consumption of Mg-rich foods [19,21], e.g., in a Western-pattern diet that is based on consuming a lot of processed food, demineralized water, and little vegetables which, in turn, themselves are low in Mg since are usually grown in soil that is poor in Mg [23]; or due to prolonged fasting or total parenteral nutrition [27];
- increased gastrointestinal loss and/or decreased absorption of Mg due to, e.g., bowel resection/bypass, short bowel syndrome, heavy alcohol consumption, or inflammatory bowel diseases (IBDs) [19,27,32];
- increased renal loss of Mg, e.g., due to diabetes mellitus, chronic metabolic acidosis, post-kidney transplantation, acute tubular necrosis, post-obstructive diuresis, or excessive volume expansion; or also in the case of rare genetic syndromes, e.g., Gitelman syndrome, or familial hypomagnesemia with hypercalciuria and nephrocalcinosis [19,24,27].

Many drugs can contribute to the development of Mg deficiency and/or hypomagnesemia. The following are the main mechanisms of medication-induced hypomagnesemia [19,22,27]: (i) promoting a shift of Mg into the cells (e.g., insulin, theophylline, metformin), (ii) increasing gastrointestinal Mg loss (e.g., metformin, laxative abuse, proton pump inhibitors); and (iii) increasing urinary Mg excretion (e.g., some antineoplastic agents, such as carboplatin; aminoglycosides, digoxin, or chronic use of loop and thiazide diuretics).

The recommended intake of Mg for adults may vary in regard to nutrition status, age, and sex. In general, the recommended daily allowance (RDA) of Mg is 310–360 mg for women and 400–420 mg for men [28,33,34]. However, for adults, the real Mg intakes in many Western countries seem to be lower than the RDA [27,33]. Therefore, Mg supplementation might be recommended for many adults, particularly for those with a high risk of developing Mg deficiency. Moreover, hypermagnesemia induced by Mg supplements is possible but extremely rare; mostly, it occurs in persons having excessive magnesium intake and/or advanced chronic kidney disease (CKD) [22,27,30,31].

## 3. Zinc

After iron, Zn is the second most common trace mineral in the body. The body has 2–4 g of Zn, of which approximately 90% is located in the bones and muscles. Only 0.1% of the total Zn is present in plasma, where it is primarily bound to albumin, and in a very small quantity, bound to metallothionein and transferrin [35,36,37].

Oysters, crabs, lobsters, beef, poultry, hard cheese, as well as seeds, nuts, and whole grains are good dietary sources of Zn [35,38,39,40]. There is no true Zn storage system in the human body; i.e., humans cannot store Zn for long. Therefore, a constant dietary intake of Zn is required to maintain normal functions [35,37,41]. 

Zn homeostasis is maintained mostly by the gastrointestinal tract and partly by other systems. Zn absorption primarily occurs in the jejunum, and it can be efficiently excreted into bile. Some of the secreted Zn is reabsorbed and undergoes enterohepatic circulation, and the daily net gastrointestinal loss is 2–4 mg/day. The Zn excretion in the kidneys is minor compared to the gastrointestinal fecal excretion; in adults, renal excretion is about 0.5 mg/day. Additional physiologic losses of Zn occur in the skin and hair [35,36,42].

Zn is crucial for body growth, development, and functioning. Zn is a part of numerous proteins, over 2500 transcription factors, and over 600 enzymes [35]. The roles of Zn in the human body can be grouped into three general functional classes: structural (component of proteins), catalytic, and regulatory. As a catalytic factor, Zn acts in enzymes from six main classes: oxidoreductases (dehydrogenases), transferases, hydrolases, lyases, isomerases, and ligases; Zn is also an important signaling mediator in the endocrine, paracrine, and autocrine systems [36]. Zn is essential in lipid, carbohydrate, and protein metabolism, in particular for the regular synthesis, storage, and secretion of insulin within pancreatic beta cells, as well as for hepatic insulin clearance and insulin sensitivity [36,42,43].

A lot of evidence has showed the antiviral, immunomodulatory and anti-inflammatory activity of Zn [39]. It impacts the survival, proliferation, and maturation of various cells involved in both adaptive and innate immunity; regulates chronic inflammation by decreasing inflammatory cytokines and antibody production; mitigates oxidative stress by upregulating antioxidant enzymes synthesis; helps to maintain natural tissue barriers like the respiratory epithelium (preventing the entry of pathogens); enhances mucociliary clearance of viruses like the coronaviruses, removing the viral particle and reducing the risk of secondary infections; potentially exhibits antiviral activity by directly preventing viral replication; and is also involved in neurotransmission, affecting learning and memory processes [24,25,36,37,39,43,44,45,46]. Interestingly, Zn has an ambivalent role: in the free form (as a transition metal), it can generate reactive oxygen species. Therefore, an excess of Zn, as well as its deficiency, may be equally harmful [46]. It was also demonstrated that Zn, being an essential trace nutrient for the intestinal microbiota, could affect the composition and function of the gut microbiome, though there are limited data regarding the metabolic mechanism [47].

Zn deficiency can present with various clinical features: growth retardation, delayed bone maturation and sexual development; hypogonadism and defective spermatogenesis in males; immune dysregulation and increased susceptibility to various infections; vision impairment (e.g., night blindness or photophobia); neural system dysfunction (including depression and anxiety); diarrhea or poor appetite; reduced taste and smell acuity; and skin lesions (erythema, erosions, etc.), hair loss, nail dystrophy and slow wound healing [35,36,37,38,41,42]. In developing countries, Zn deficiency is considered one of the main causes of morbidity [41]. Zn deficiency is associated with the development and/or worsening of many diseases: alcoholic hepatitis and other liver diseases, cancer cachexia, chronic obstructive pulmonary disease (COPD), IBD, obesity, osteoporosis, and sarcopenia [36,41].

It appears that Zn homeostasis and VitD functioning are linked. Zn can regulate the expression of vitamin D-dependent genes via contributing to VDR conformational changes and intensifying the activity of specific vitamin D-dependent promoters; therefore, Zn is considered an essential cofactor for VitD activity [35,44]. On the other hand, VitD can directly augment the expression of Zn transporters, such as ZnT10; an upregulation of the ZnT10 protein allows Zn to migrate out of the cytosol, and increased concentrations can be available for extracellular use [35,44]. However, it was also hypothesized that, by improving the intracellular Zn concentrations, VitD can mitigate oxidative damage; and increased intracellular oxidative stress is known to contribute to the development of many pathologies, such as cardiovascular disease, neurological disorders, cancer, diabetes mellitus or ischemia [46].

The Zn levels can be measured in the whole blood, plasma, serum, urine, and hair [37,48]. The plasma (or serum) Zn levels, although their testing is accessible in many facilities, do not necessarily represent the body’s total Zn content. This is partly due to the variable amount of Zn in the intracellular compartments, including nucleic acids, which cannot be measured in plasma samples [35]. In addition, due to the substantial intra-individual variability in the plasma Zn concentration, this biomarker should be used with caution and is best suited to estimating population status [42]. However, plasma or serum Zn assessment remains the most widely used test to confirm clinical Zn deficiency and to monitor the adequacy of provision [36,37].

Normal serum Zn values are in the range of 80–100 µg/dL (12–15 µmol/L) [37]. Of note, the plasma/serum Zn levels decrease during the inflammation-associated acute-phase response. Therefore, simultaneous determinations of *C*-reactive protein (CRP) or other markers of the acute-phase response might also be useful in clinical practice. The plasma Zn decreases significantly whenever the CRP exceeds 20 mg/L, complicating the interpretation of the results [36]. In addition, serum albumin determination is also important, since hypoalbuminemia (due to dilution, inflammation, or advanced liver disease) can artificially lower the serum Zn levels [36,37,40].

Zn deficiency is common, especially in developing countries; however, it is a potentially underdiagnosed disorder [35]. Inadequate intake, malabsorption, increased requirements, increased losses and/or impaired utilization are the main mechanisms of Zn deficiency, and various risk factors can contribute to its development [21,35,36,37,39,41,42]:

- IBD, bariatric surgery, chronic pancreatitis, liver diseases, short bowel syndrome, or cystic fibrosis;
- phytate-rich diet, particularly in vegetarian populations;
- starvation;
- eating disorders (e.g., anorexia nervosa or bulimia);
- chronic parenteral nutrition with low or without any Zn supplementation;
- obesity;
- aging;
- infancy, childhood, adolescence, pregnancy and lactation (due to increased requirements for Zn);
- drinking excessive amounts of alcohol (due to the tendency of decreased Zn absorption, increased Zn excretion abilities, and less Zn intake);
- diabetes mellitus;
- renal disease resulting in chronic renal failure or prolonged renal replacement therapy;
- hypercatabolic conditions, such as burns, trauma and sepsis;
- drugs that increase Zn excretion, e.g., thiazide diuretics;
- drugs, that reduce intestinal Zn absorption, e.g., some antibiotics (particularly quinolones and tetracyclines);
- iron, contained in daily supplements, and cadmium, which is increasing in the environment and in food (both metals are capable of decreasing Zn absorption);
- rare hereditary diseases, e.g., acrodermatitis enteropathica, caused by an autosomal recessive mutation in the gene coding for the ZIP4 transporter, or a sickle cell disease.

Regarding the daily Zn intake, various recommendations exist. Most of them, however, suggest a RDA of elemental Zn in the range from 7 to 20 mg for adults; a higher intake is recommended for pregnant and lactating women, as well as for those with a high daily phytate intake [35,36,40,42,48,49]. Zn toxicity is a rare condition; nevertheless, to minimize the risk of toxicity, the upper tolerable limit of 40 mg/day for many adults should not be exceeded [35,37,40]. However, in the case of Zn deficiency, therapeutic Zn doses of 50 or 75 mg/day might be used (with periodical serum Zn level monitoring) [40].

## 4. Vitamin K

VitK, a fat-soluble vitamin, is actually a group of molecules that share a common methylated naphthoquinone ring but with different side chains. Naturally, VitK exists in two main forms: K1 (=phylloquinone) and K2 (=menaquinones). Vitamin K2 (VK2) comprises a collection of different compounds known as menaquinone-n (MK-n), with variation in the length of the unsaturated isoprene side chain in the molecule (n means the number of isoprene units), ranging from MK-2 to MK-15; MK-4, MK-7, and MK-9 are the most studied menaquinones [50]. Vitamin K3 (menadione) is a synthetic water-soluble form of the vitamin and is converted into VK2 by the liver. Vitamin K3 is primarily used for industrial and research purposes [51]. Other VitK forms—K4 and K5—also exist, but they are only available in a synthetic form [52]. 

Vitamin K1 (VK1) is abundant in green leafy vegetables, which are the main dietary source of VitK; VK1 is also produced in algae (via photosynthesis) [50,51,53]. VK2 is synthesized by the human intestinal microbiota and the primary dietary sources of VK2 are of microbial origin, commonly found in fermented foods such as cheese, curds, cream, sour cream, butter, and fermented soybean product *natto*, as well as in eggs, chicken, ham, and animal livers [51,53]. In the Western diet, the majority of the VitK intake occurs in the form of VK1. However, VK2 has a higher bioavailability than VK1 and may therefore be at least as important for its bioactivity [54].

The absorption of VitK mainly occurs in the jejunum and ileum, and minimally in the colon; this process requires the presence of dietary fat and the action of pancreatic juices in the duodenum for solubilization within mixed micelles [55]. VK2 is easier to absorb and has a longer half-life than VK1, which is particularly evident for long-chain menaquinones with high lipophilicity [56]. VK1 is mostly taken up by the liver, where it is used to activate coagulation factors. Long-chain VK2 is more lipophilic and, due to the long plasma half-life, more efficaciously penetrates extra-hepatic tissues (e.g., blood vessels and bones) and activates specific proteins there [54]. 

The main role of VitK in the human body is to be a cofactor for the enzyme γ-glutamyl carboxylase (GGCX) and to aid in the post-translational carboxylation of protein-bound glutamate residues into γ-carboxyglutamate (GLA) [52]. Humans, like many animals, are able to regenerate VitK via the “vitamin K cycle”, which is tied to that carboxylation. In brief, VitK is reduced to the biologically active vitamin K hydroquinone. Via the process of the γ-carboxylation of vitamin K-dependent proteins (VKDPs), catalyzed by GGCX, VitK hydroquinone is oxidized into VitK epoxide. Subsequently, in a two-step reduction catalyzed by vitamin K epoxide reductase and vitamin K reductase, VitK epoxide is converted back into VitK hydroquinone [54]. There are 17 VKDPs that regulate blood coagulation, bone metabolism, and arterial calcification [53]. However, regarding biological functions of VitK, a novel mechanism for the regulation of the transcription of target genes has been found: VK2 was shown to be capable of binding to and activating the nuclear steroid and xenobiotic receptor (SXR), which, in turn, could induce expression of osteoblastic marker genes, such as alkaline phosphatase and osteoprotegerin, extracellular matrix-related genes, and collagen accumulation in osteoblastic cells [57].

It is well known that VitK is involved in the synthesis of clotting factors in the liver and in the enabling (via carboxylation) of clotting proteins like factor II, VII, IX, and X to function in blood clotting [51], and VitK deficiency results in the impairment of blood coagulation and hemorrhagic disorders. However, VitK is prioritized for blood coagulation at the expense of bone health [21]. Indeed, VitK, mainly VK2, has a crucial role in the promotion of osteogenesis and prevention of calcification [50]. 

In bones, VitK acts via several pathways. Firstly, VitK helps to bind Ca in bones by the formation of GLA residues on osteocalcin (OC). OC, the predominate non-collagenous protein in bone, is a VitK-dependent protein and is synthesized only in osteoblasts [54,58,59]. It is well known that the carboxylation of GLA domains helps the proteins (containing GLA domains) in Ca binding, and OC also has GLA domains [59]. After vitamin K-dependent carboxylation, OC contributes to both the formation of hydroxyapatite crystals in bone and the inhibition of bone mineralization, thereby preventing over-mineralization [54]. VK2 has also been shown to induce OC synthesis [60]. Secondly, VK2 stimulates the differentiation of osteoblasts and inhibits osteoclastogenesis [57,61]. Thirdly, VK2 is involved in the activation of BMP-2 (bone morphogenetic protein-2) and Wnt/β-catenin signaling, being important in bone formation and homeostasis [61]. Fourthly, VK2, in particular MK-4, can decrease cyclooxygenase-2 expression as well as prostaglandin E2 production during osteoclast development, potentially contributing to the inhibition of bone resorption [58]. In addition, VitK can protect bone tissue against reactive oxygen species produced by osteoblasts [54].

VitK can also be important to the prevention of vascular calcification (VC). VC is a result of the smooth muscle cells behaving like osteoblasts, allowing synthesis of hydroxyapatite crystals, similar to the remodeling process in bone. This occurs in the media layer and, as a result, the artery becomes stiff and unable to dilate or constrict [52]. The underlying pathology occurs due to a lack of activation of the inhibitory proteins, e.g., matrix GLA protein (MGP), which can prevent the mineralization of vessels. Activated (by carboxylation) MGP acts as a natural inhibitor of arterial calcification, thereby contributing to maintaining healthy arteries. MGP is believed to inhibit bone morphogenetic proteins, namely, BMP-2 and BMP-4; these BMPs are proteins that enhance the VC [51,52,61]. The deficiency of VitK results in the synthesis of inactive MGP, causing increased calcification of the blood vessels [60]. Therefore, VitK may be considered protective not only against osteoporosis but also against VC and associated cardiovascular disease, as well as heart valve calcification [51,52,53,54,61].

VitK was also demonstrated to have anti-inflammatory activity and to function as a potent antioxidant [51]. VitK deficiency is associated with many diseases, including osteoarthritis, obesity, COPD, sarcopenia, diabetes mellitus, fat malabsorption, CKD, cancers, dementia, cognitive impairment, mobility disability, and frailty [51,54,60,62].

VitD appears to have some synergistic effects with VitK. Calcitriol upregulates MGP and OC expression, and VitK is required for the proper γ-carboxylation of these proteins [54,57]. Another area of VitD and VitK cooperation is inflammation, since both vitamins are known to decrease the production of certain proinflammatory cytokines [57]. Thus, VitK increases vitamin D’s beneficial effect on bone mineralization [57], as was shown in some clinical trials, as reviewed elsewhere [60,63]. Interestingly, it was even suggested that for osteoporosis treatment, not only VitD plus Ca but also additional supplementation with VK2 plus the amino acid taurine might be recommended [59]. In COPD or COVID-19 infection, combined supplementation with VitD and VitK might be used, potentially suppressing the impairment of the lungs [50,54]. In addition, VitD and VitK can cooperate in preventing arterial stiffness and vascular calcification [54,60]. Moreover, VitK supplementation may offer a defense against any unfavorable consequences of Ca supplementation [60]. In summary, VitD and VitK cooperation is useful in combating the “calcium paradox”—the resultant increase in arterial calcification and decrease in the Ca content of bone when Ca metabolism is impaired [52]. Of note, if was suggested that excessive amounts of VitD can augment VitK requirements, since direct stimulation of the synthesis of VKDPs can lead to a relative VitK deficiency [57].

Interestingly, VitK and VitD can have some metabolic overlap at the cellular level. SXR, which can be activated by VK2, is able to crosstalk with VDR, and this SXR–VDR interaction can suppress the activity of the VDR-mediated CYP24 promoter; this results in decreased CYP24-mediated hydroxylation, i.e., reduced catabolism of calcitriol (and also reduced deactivation of 25OH-D). On the other hand, calcitriol can enhance the reductive recycling of VK2. Therefore, VitK and VitD can mutually intensify each other’s metabolism [57].

Laboratory assessment of the VitK status by direct measurement of the VitK levels (e.g., in serum) is available, but it is difficult and demonstrates high variability; therefore, a deficiency of VitK is usually detected by functional assays [53,64], e.g., the prothrombin time (PT). VitK deficiency classically leads to elongated PT and bleeding in the setting of minor or absent trauma [64]. Another serum marker of the VitK levels is known as “Protein Induced by Vitamin K Antagonism or Absence” (PIVKA). The PIVKA levels usually increase in the case of VitK deficiency [64]. Undercarboxylated coagulation factor II, referred to as “Protein Induced by Vitamin K Antagonism or Absence II” (PIVKA-II), is a less commonly used but potentially very accurate marker, reflecting the hepatic VitK status [54,63]. The inactive form of the calcification inhibitor MGP, i.e., dephosphorylated undercarboxylated MGP, and undercarboxylated OC can be used as biomarkers of an extra-hepatic VitK status [54]. However, as there is no agreed standard, the VitK status should be evaluated using a combination of biomarkers combined with dietary VitK intake assessment [36,64].

VitK deficiency can be caused by various factors, with insufficient dietary intake being the most important [54]. Similarly to Mg and Zn deficiency, various diseases resulting in malabsorption (IBD, chronic pancreatitis, etc.) might also play an important role. VitK deficiency is very common in CKD, worsens as CKD progresses, and is associated with a low dietary intake of VitK in hemodialysis patients [53,63]. Anticoagulants, proton pump inhibitors, phosphate binders, and calcimimetics may reduce VitK and interfere with its actions [53], as well as antibiotics that inhibit the intestinal flora and thus reduce the bacterial production of VitK [53,54,63].

The RDA for the VitK intake varies per country and is usually set as 50–120 μg per day [36,54,65]. For treatment, VK1 in doses from 1 to 2 mg is usually suggested [66]. Obviously, the VitK intake should be closely monitored in subjects at a higher risk of hypercoagulation due to its role in blood clotting [61].

## 5. Discussion

The deficiency of various nutrients in many countries remains a serious problem even in the 21st century. A plethora of risk factors were shown to contribute to the development of various deficiencies. The current review outlined the main risk factors regarding Mg, Zn and VitK deficiency. It appears that these deficiencies share many common risk factors with low VitD status (VitD insufficiency or deficiency), e.g., intestinal malabsorption, unhealthy diet, aging, or increased requirements for certain nutrients during some periods of life [67]. However, in clinical practice, some nutrient deficiencies, particularly in their mild forms, may remain undiagnosed and this may lead to undesirable results, e.g., worsening or progression of certain chronic diseases. On the other hand, a deficit of Mg, Zn or VitK is associated with, and can even contribute to, the occurrence or progression of the same illnesses as in the case of VitD deficiency, e.g., diabetes mellitus, cardiovascular diseases, recurrent respiratory infections, depression, musculoskeletal diseases, obesity, etc. In addition, evidence showed that VitD could have a significant influence on the metabolism and/or activity of Mg, Zn, and VitK, and vice versa. It could be hypothesized that different intracellular levels of nutrients like Mg or, particularly, Zn might be the clue to explaining the nature of different personal vitamin D response indexes [17].

Overall, from the clinical point of view, VitD appears to have a beneficial synergism with the studied nutrients. Therefore, once low VitD is diagnosed, searching for possible deficiencies of other nutrients might be useful. In case the assessment of the Mg, Zn or VitK status is not currently available but the deficiency of one or several nutrients is highly possible, for some patients it might be reasonable to recommend additional supplementation with prophylactic doses of the nutrients. For adults, it might be 300–400 mg of Mg, 15–20 mg of Zn, and/or 90–120 μg of VitK. These doses would be considered “safe” for many patients. The rationality behind similar recommendations is obvious: since a patient is highly suspected to have a deficiency of one or several nutrients, the current nutrient status is expected to improve with the help of appropriate supplementation. This amelioration of the nutrient status likely results in the improvement of the patient’s health, e.g., in better control of chronic disease(s), and in a lower burden for healthcare systems. A higher increase in the serum 25OH-D levels might also appear, but this should not be the main target when supplementation with Mg, Zn or VitK is started. One of the main goals of this supplementation, in particular when it is combined with VitD supplementation, should be to achieve higher intracellular levels of calcitriol and to increase the activity of calcitriol in various cells, expecting the improvement of many physiological processes; the latter does not necessary correlate with better serum 25OH-D levels. It should be emphasized that the doses of Mg, Zn and VitK suggested in this review for supplementation are just general considerations. The final decision in certain clinical situations regarding supplementation must be made by the physician based on the patient’s age, dietary patterns, comorbidities, and other factors. 

It should be noted that the purpose of the current review was not to criticize the available VitD guidelines [2,3,4,5,6,7,8,9,10,11], since those guidelines were developed against the background of the evidence available at that time. Therefore, it could be speculated, when more evidence (particularly from clinical trials) arrives, that future VitD guidelines will include sections not only on Mg but also on such nutrients as Zn or VitK supplementation.

## 6. Conclusions and Future Perspectives

The deficiency of nutrients like Mg, Zn, and VitK, as well as VitD, which in many cases is undiagnosed, is still a huge problem in clinical medicine. Undoubtedly, for a better understanding of the crosstalk among the aforementioned nutrients, more research, including clinical trials, is needed. Nevertheless, the accumulated evidence showed the presence of various relationships of VitD with Zn, Mg and VitK—in regard both to metabolism and to activity. Better education of the medical community about the risk factors for the deficiency of these nutrients, its consequences for human health, the diagnostic possibilities, as well as measures for improving the nutrients’ status is also needed. These means are expected to help many patients in improving the control of their diseases as well as their quality of life.
